# AI Predictive Model of Mortality and Intensive Care Unit Admission in the COVID-19 Pandemic: Retrospective Population Cohort Study of 12,000 Patients

**DOI:** 10.2196/70674

**Published:** 2025-07-10

**Authors:** Jose Manuel Ruiz Giardin, Óscar Garnica, Nieves Mesa Plaza, Juan Víctor SanMartín López, Ana Farfán Sedano, Elena Madroñal Cerezo, Miguel Ángel Duarte Millán, Aida Izquierdo Martínez, Luis Rivas, Marta Rivilla, Alejandro Morales Ortega, Begoña Frutos Pérez, Cristina De Ancos Aracil, Ruth Calderón, Guillermo Soria Fernandez, Jorge Marrero Francés, David Bernal Bello, Jose Ángel Satué Bartolomé, María Toledano Macías, Sara Piedrabuena García, Marta Guerrero Santillán, Rafael Cristóbal, Belen Mora, Laura Velázquez Ríos, Vanesa García de Viedma, Paula Cuenca Ruiz, Ibone Ayala Larrañaga, Lorena Carpintero, Celia Lara, Alvaro Ricardo Llerena, Virginia García Bermúdez, Gema Delgado Cárdenas, Paloma Pardo Rovira, Elena Tejero Sánchez, Maria Jesús Domínguez García, Carolina Mariño, Cristina Bravo, Ana Ontañon, Mario García, Jose Ignacio Hidalgo Pérez, Santiago Prieto Menchero, Natalia González Pereira, Sonia Gonzalo Pascua, Jorge Tarancón Rey, Luis Antonio Lechuga Suárez, FUENCOVID

**Affiliations:** 1 Medicina Interna-Infecciosas Hospital Universitario de Fuenlabrada Fuenlabrada Spain; 2 CIBERINFECT Centro de Investigación Biomédica en Red Madrid Spain; 3 Departamento de Arquitectura de Computadores Universidad Complutense de Madrid Madrid Spain; 4 Medicina Interna Hospital Universitario de Fuenlabrada Fuenlabrada Spain; 5 Urgencias Hospital Universitario de Fuenlabrada Fuenlabrada Spain; 6 Farmacia Hospitalaria Hospital Universitario de Fuenlabrada Fuenlabrada Spain; 7 Laboratorio Clínico Hospital Universitario de Fuenlabrada Fuenlabrada Spain; 8 Sistemas Hospital Universitario de Fuenlabrada Fuenlabrada Spain; 9 Hospital Universitario de Fuenlabrada Fuenlabrada Spain

**Keywords:** SARS-CoV-2, COVID-19, death, mortality, intensive care unit, population study, predictive model, artificial intelligence, random forest

## Abstract

**Background:**

One of the main challenges with COVID-19 has been that although there are known factors associated with a worse prognosis, clinicians have been unable to predict which patients, with similar risk factors, will die or require intensive care unit (ICU) care.

**Objective:**

This study aimed to develop a personalized artificial intelligence model to predict the patient risk of mortality and ICU admission related to SARS-CoV-2 infection during the initial medical evaluation before any kind of treatment.

**Methods:**

It is a population-based, observational, retrospective study covering from February 1, 2020, to January 24, 2023, with different circulating SARS-CoV-2 viruses, vaccinated status, and reinfections. It includes patients attended by the reference hospital in Fuenlabrada (Madrid, Spain). The models used the random forest technique, Shapley Additive Explanations method, and processing with Python (version 3.10.0; Python Software Foundation) and scikit-learn (version 1.3.0). The models were applied to different epidemic SARS-CoV-2 infection waves. Data were collected from 11,975 patients (4998 hospitalized and 6737 discharged). Predictive models were built with records from 4758 patients and validated with 6977 patients after evaluation in the emergency department. Variables recorded were age, sex, place of birth, clinical data, laboratory results, vaccination status, and radiologic data at admission.

**Results:**

The best mortality predictor achieved an area under the receiver operating characteristic curve (AUC) of 0.92, sensitivity of 0.89, specificity of 0.82, positive predictive value (PPV) of 0.35, and mean negative predictive value (NPV) of 0.98. The ICU admission predictor had an AUC of 0.89, sensitivity of 0.75, specificity of 0.88, PPV of 0.37, and NPV of 0.98. During validation, the mortality model exhibited good performance for the nonhospitalized group, achieving an AUC of 0.95, sensitivity of 0.88, specificity of 0.98, PPV of 0.21, and NPV of 0.99, predicting the death of 30 of 34 patients who were not hospitalized. For the hospitalized patients, the mortality model achieved an AUC of 0.85, sensitivity of 0.86, specificity of 0.74, PPV of 0.24, and NPV of 0.98. The model for predicting ICU admission had an AUC of 0.82, sensitivity of 1.00, specificity of 0.59, PPV of 0.05, and NPV of 1.00. The models’ metrics presented stability along all pandemic waves. Key mortality predictors included age, Charlson value, and tachypnea. The worse prognosis was linked to high values in urea, erythrocyte distribution width, oxygen demand, creatinine, procalcitonin, lactate dehydrogenase, heart failure, D-dimer, oncological and hematological diseases, neutrophil, and heart rate. A better prognosis was linked to higher values of lymphocytes and systolic and diastolic blood pressures. Partial or no vaccination provided less protection than full vaccination.

**Conclusions:**

The artificial intelligence models demonstrated stability across pandemic waves, indicating their potential to assist in personal health services during the 3-year pandemic, particularly in early preventive and predictive clinical situations.

## Introduction

At the end of 2023, the COVID-19 pandemic had produced more than 766 million confirmed infections (laboratory-confirmed tests, such as polymerase chain reaction or antigen test), with 7 million deaths worldwide. In Spain, 13 million cases have been confirmed, with 121,000 deaths. In Madrid, 2,004,209 were infected, with 21,246 deaths [1,2].

The Hospital Universitario de Fuenlabrada (HUF) is the reference hospital for a population close to 220,000 inhabitants in the south of Madrid. This means that HUF is the only hospital where people from Fuenlabrada are admitted due to COVID-19. It has attended to more than 5000 hospitalized patients for COVID-19 with a mortality rate changing depending on the vaccination status and the SARS-CoV-2 variants.

Massive vaccination campaigns have become the primary strategy against the pandemic. Messenger RNA vaccines and vaccines based on defective viral vectors have been the most frequently administered vaccines in Spain [3-5]. Between December 27, 2020, and October 14, 2021, the National Health System notified that 90% of the target population received at least 1 dose, and 88% completed the regimen, more than 40,700,000 people [6].

Physicians are aware of many factors associated with worse prognosis (immunosuppression, diabetes, malignancy, gastrointestinal symptoms such as nausea, vomiting, and abdominal pain, respiratory symptoms such as shortness of breath and chest pain, invasive mechanical ventilation, HIV status, and metabolic acidosis were some of the most important) and higher mortality (older age, male sex, diabetes, hypertension, pneumonia, and end-organ failure) [7,8]. However, the reasons why patients with similar demographics, vaccine status, and risk factors have a different evolution are still unknown. About these, previous models for predicting COVID-19 mortality were often inadequate for individual-level predictions due to several factors such as poor performance in external validations, high variability in data and methodological bias, inconsistent performance in older populations, dynamic changes in the pandemic context, heterogeneity in study populations, and technological and methodological limitations: Early models often relied on single prediction techniques and lacked robust validation, leading to limited generalizability and applicability in diverse clinical settings.

The aim of this observational, retrospective, cohort study is to develop at-admission predictive clinical models for mortality and intensive care unit (ICU) admission using machine learning (ML) techniques based on the clinical, epidemiological, and analytical characteristics of the patients the first moment they consult in the emergency department due to SARS-CoV-2 infection and before receiving any treatment. This model has been created only considering those patients with COVID-19 who have needed hospital admission during the COVID-19 pandemic (3 years). Later, the model has been validated.

The artificial intelligence (AI) approach to predicting COVID-19 mortality or ICU admission has proven to be a significant improvement over previous clinical tools due to its ability to integrate and analyze a large number of complex and multimodal variables, allowing for more efficient management of patients and resources in hospital settings.

ML techniques improve upon traditional models for several reasons. They enable the creation of personalized, predictive models that can analyze complex, high-dimensional medical data and identify patterns that conventional statistical models might miss. ML can handle large amounts of diverse data and uncover nonlinear relationships between variables and outcomes that traditional models might overlook. ML models can adapt to evolving data distributions, unlike conventional models, which may need manual recalibration as conditions change. This study uses the Shapley Additive Explanations (SHAP) method to determine which variables impacted the prediction the most. Traditional models typically provide fixed coefficients, but ML methods offer flexible, data-driven insight into relevant features.

The study’s importance is fourfold: (1) the highly relevant classification metrics obtained after model validation; (2) it covers a population of 220,000 inhabitants in one of the areas with a large number of infections in Madrid; (3) it is validated using all infected patients who were attended at the hospital, either admitted or discharged; and (4) patients have been infected with all the circulating SARS-CoV-2 variants during the pandemic.

## Methods

### Ethical Considerations

In 2020, the clinical research ethics committee of HUF approved the “Database of COVID-19 Patients Treated at Hospital Universitario de Fuenlabrada (FUENCOVID)” project (approval 20/39). Given the retrospective nature of the study and the large number of patients included, obtaining individual informed consent was not feasible. The study relied solely on data previously collected during routine clinical care, and all data were fully anonymized prior to analysis. According to the policies of the ethics committee of HUF, and considering that the study involved no direct patient contact or additional risk to participants, the ethics committee determined that informed consent was not required. No patients received any kind of compensation.

### Study Design

This population-based, observational, and retrospective study covers 3 years of evolution since the beginning of the COVID-19 pandemic, with all the patients attending the only reference hospital in the city of Fuenlabrada (Madrid, Spain). All the processing has been performed using Python (version 3.10.0; Python Software Foundation) and scikit-learn (version 1.3.0) [9].

### Data Collection

The objective was to create a large-scale database containing anonymized, structured, and nontraceable medical information of patients with SARS-CoV-2 infection who were treated at the hospital, whether admitted or discharged. The database included records of 11,975 patients.

Baseline epidemiological, clinical, laboratory, therapeutic, vaccine status, and radiological data were collected at admission for all patients. Additional information on clinical evolution and COVID-19 treatment during hospitalization was also recorded, amounting to over 700 variables. The Integrating Biology and Bedside data warehouse was used, enabling standardized data acquisition and incorporating features such as postdischarge disease evolution and mortality records. Deaths occurring during hospitalization or within 30 days after discharge were considered COVID-19–related.

### Data Preprocessing

In addition to standard preprocessing procedures, several additional steps were taken: (1) categorical features were encoded while preserving their order relation, (2) out-of-range values in features were removed, and (3) each patient’s initial date was determined as their first contact date with the hospital attention, and all subsequent dates, including vaccination dates, were recalculated relative to this initial date.

Missing values in the dataset were handled using the separate class method. The missing values of each feature are randomly distributed across the records and neither introduce any uninformed bias of the measurement or recording procedure nor provide any insight into the target variable in the prediction [10]. The datasets contained a high number of features relative to the number of instances, which could potentially introduce noise and reduce accuracy. To address this, recursive feature elimination with k-fold cross-validation was applied. This method selected the minimum subset of features that achieved the best model accuracy, avoiding overfitting by using cross-validation [11].

### Datasets

Two datasets were created for training and testing ML models using data from patients hospitalized from February 2020 to April 2022. The first dataset (exitus dataset) focused on predicting patient mortality caused by SARS-CoV-2 infection, while the second dataset (ICU dataset) aimed to predict admission to the ICU during the hospital stay. Both datasets experienced class imbalance, which could negatively impact model performance. Two oversampling techniques—synthetic minority oversampling technique and SMOTETomek [12]—were tested to address this situation. Both are based on generating new synthetic samples (data augmentation) of the minority class along the segment connecting actual minority class samples. SMOTETomek adds a cleaning step by removing the samples in the majority class closest to those in the minority class. Both techniques were used to evaluate whether new synthetic samples could improve the model performance. Since there are no standardized tests for assessing the utility of synthetic data, we relied on the improvement of the models’ performance metrics to evaluate their usefulness. Models’ metrics did not improve. Given the good performance of the models without synthetic data and concerns regarding the realism of the synthetic data when applied to complex, real-world medical scenarios [13], we decided not to use any data augmentation technique.

The models were validated using separate datasets: a validation dataset of hospitalized patients who were ultimately admitted from April 2022 to January 2023 and a validation dataset of nonhospitalized (discharged) patients between February 2020 and January 2023. All datasets share the same features and preprocessing procedures, including only the features known at admission.

### Random Forest

Random forest (RF) is a supervised ML technique that uses an ensemble of decision trees for classification and regression. It combines the outputs of multiple trees to provide a probability of positive classification for a given input based on the ratio of decision trees that return a positive classification. The higher the probability, the stronger the confidence that the patient’s data effectively align with patterns associated with a positive outcome [14]. A threshold value should be provided so that a binary yes or no outcome is returned. If risk stratification is required rather than a binary outcome, the probability scores generated by the RF model can be used. RF is favored for three reasons: (1) it achieves accuracy comparable to deep learning models in applications without strong temporal or spatial correlation among data [15], (2) it offers better interpretability compared to deep learning models [16], and (3) previous studies have ranked RF among the top ML techniques for biomedical prediction models [17]. The architecture of an RF model is determined by several hyperparameters, which are empirically tuned using a 10-fold cross-validation grid search to optimize performance and minimize model size [18].

### Metrics and Interpretability

In data with normal distributions, the mean and SD are used, while for nonnormal distributions, the median and IQR are used. Due to the imbalance in the dataset, the accuracy metric is not recommended, as it focuses on improving the true positive rate of the majority class. To address this, the area under the receiver operating characteristic curve (AUC) is chosen as the metric to optimize during model training.

The SHAP technique is used to interpret the importance of features in predictions. SHAP is an agnostic technique that uses Shapley values from coalitional game theory to explain the significance of features in predicting an instance’s outcome [19]. SHAP calculates the difference in model output by including or excluding individual features. This process yields a Shapley value for each feature, providing insights into both its contribution and its impact on the model’s predictions. The SHAP method assesses the contribution of each feature value to the classification, taking into account the removal of the prevalence. This knowledge becomes crucial for clinicians to identify the most influential variables that significantly affect the prediction. It is essential to highlight that the significance of SHAP values does not rely solely on their magnitude, as lower SHAP values do not necessarily imply less importance. If the difference between the SHAP base value (prevalence) and the classification threshold is small, even a low SHAP value for a feature can contribute to a positive classification.

Furthermore, the SHAP value for a specific instance and feature can be decomposed into the interactions of the feature with all other feature values for that instance. The SHAP values of these interactions quantify the importance of combinations of feature values for classifying an instance.

## Results

### Dataset Characterization

The analysis of hospitalized patient records used for model training and testing finds that 2729 (54.6%) were male with an average age of 61.0 (SD 16.7) years, average BMI of 29.3 (SD 5.2), and average disease evolution time of 7.2 (SD 4.2) days. The remaining 2269 (45.4%) were female with an average age of 62.1 (SD 19.2) years, average BMI of 30.2 (SD 6.3), and average disease evolution time of 7.4 (SD 4.5) days. The median time to death since hospital admission was 11.9 (IQR 5.6-18.2) days, and the median time to ICU admission was 2.8 (IQR 0.8-4.8) days.

**Figure 1:**
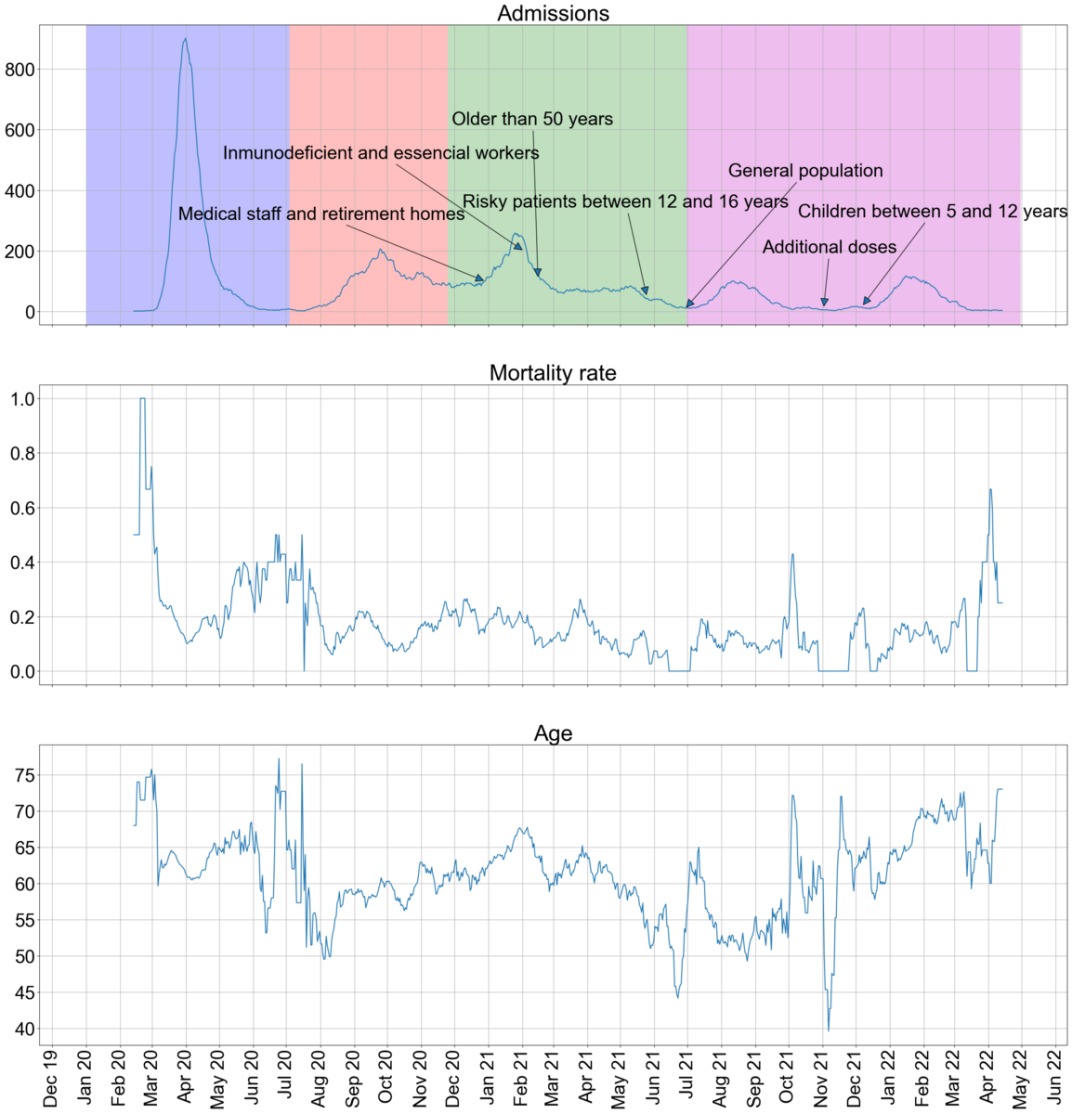
Moving 14-day sliding window average of the evolution in the number of admissions, mortality rate, and age (in years) between January 2020 and April 2022. Background colors represent the dominant SARS-CoV-2 variants over time: blue is Wuhan, red is the Alpha variant, green is the Delta variant, and magenta is the Omicron variant. The arrows mark the starting dates for the vaccination of different collectives.

Figure 1 [20,21] illustrates the trends in admissions, mortality rate, and age over the study period. Data have been smoothed using a moving 14-day sliding window average to filter high-frequency stochastic behavior. Spain has experienced 7 waves of SARS-CoV-2 infections, leading to 7 hospital admissions waves in HUF [20].

The exitus dataset contains 4710 patients with 126 features known at admission per patient, with 472 (10%) patient deaths during admission or in the first 30 days after discharge. The ICU dataset has 4998 patients with 124 features per patient and 409 (8%) ICU admissions. The prevalence of COVID-19 infections for each SARS-CoV-2 main variant is presented in Table 1.

**Figure 2:**
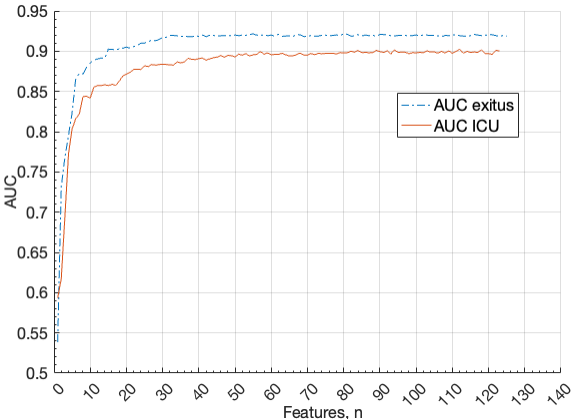
AUC of the random forests for the different number of variables for death (exitus) and ICU admission (ICU). AUC: area under the receiver operating characteristic curve; ICU: intensive care unit.

Feature selection using recursive feature elimination with k-fold cross-validation and AUC resulted in 60 selected features for both mortality and ICU admission prediction. Figure 2 illustrates how models’ AUCs remain stable, with values equal to or greater than 0.90, up to 60 features. Note that while the number of features is the same, the selected features differ depending on whether the prediction is for mortality or ICU admission. Finally, the exitus dataset was reduced to 35 features, while the ICU dataset contained 61 features.

The validation dataset (April 2022 to January 2023) contains 240 hospitalized patients with 43 deaths and 5 ICU admissions and 6737 nonhospitalized patients with 34 deaths within 30 days after discharge. Patients who died more than 30 days after discharge were eliminated from the validated dataset to avoid the class noise [22]. The datasets’ missingness is random and unrelated to the outcomes. Of all the patients predicted by our model to die, 21%-25% actually did. Conversely, among the patients predicted by our model to survive, 98%-99% did not die.

**Table 1:** Number of patients and proportion of positive infection cases (prevalence) per dataset for the 4 variants.

Variant	Exitus dataset			ICU^a^ dataset	
	Patients (n=4710), n (%)	Prevalence	Patients (n=4998), n (%)		Prevalence
Wuhan	1721 (36.5)	0.11	1836 (36.7)		0.06
Alpha	924 (19.6)	0.09	983 (19.6)		0.08
Delta	1293 (27.4)	0.10	1377 (27.5)		0.10
Omicron	772 (16.4)	0.09	802 (16)		0.10

^a^ICU: intensive care unit.

### Model Training and Test

The best RF parameters are (1) 600 estimators, (2) Gini metric, (3) maximum depth of 20, and (4) 8 features per tree. These values have been obtained using Optuna, a framework for searching optimal hyperparameters using Bayesian optimization.

**Figure 3:**
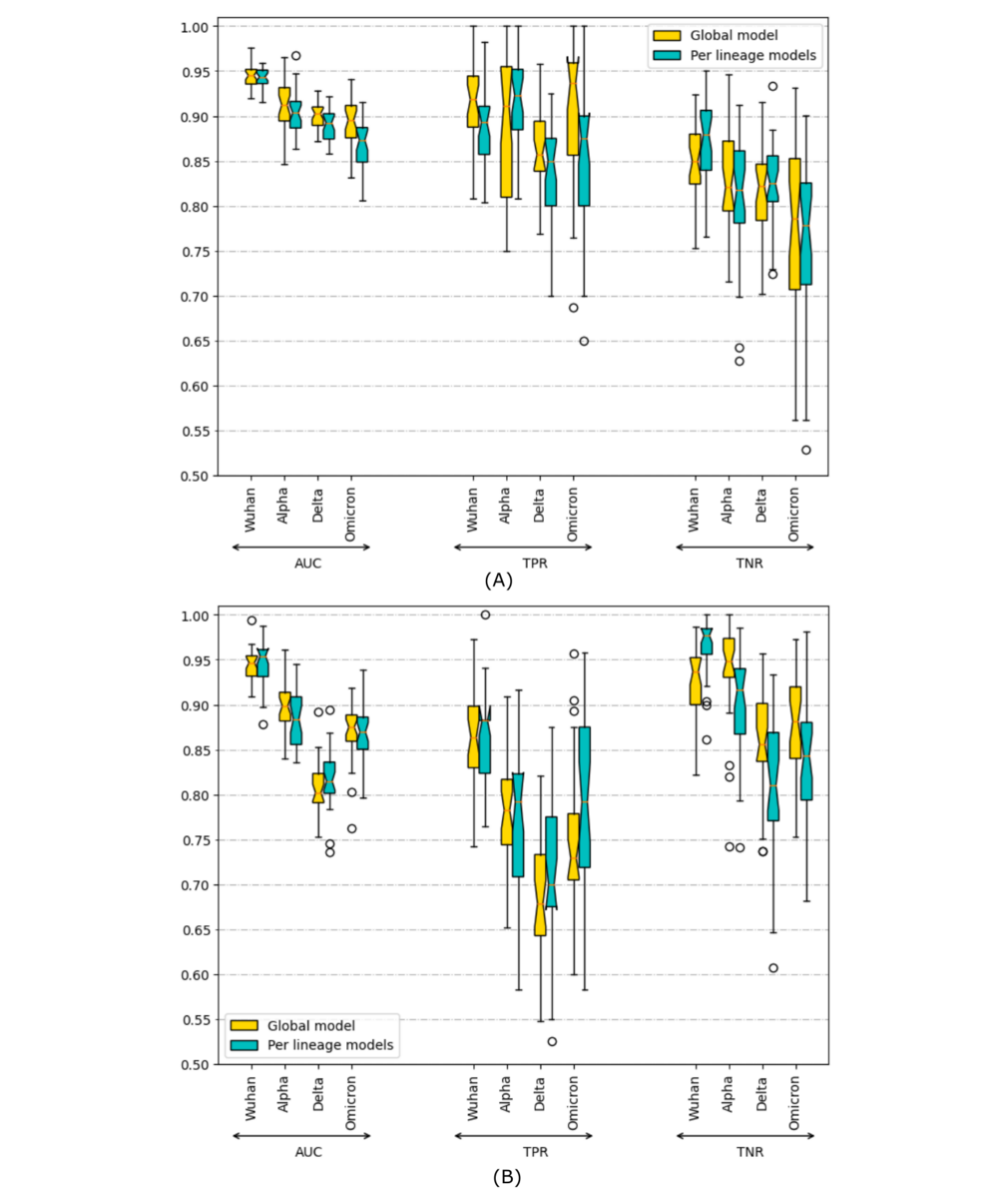
Comparison of the AUC, TPR, and TNR metrics values for the 4 predominant variants of SARS-CoV-2 for the per-variant and global models for both datasets. Abscissas represents the value of the metrics, and the ordinate represents the 4 variants grouped by metric. Green bars for each variant represent the metric of the per-variant model of that variant; yellow bars represent the metric of the global model for that variant. (A) Mortality predictor model and (B) ICU admission predictor metrics. AUC: area under the receiver operating characteristic curve; TNR: true negative rate; TPR: true positive rate.

The evolution of SARS-CoV-2 variants with different clinical metrics raises the question of whether predictive models per variant can improve prediction performance. To investigate this, we created 5 models per dataset: 1 global model trained with all variants (global) and 4 per-variant models (Wuhan, Alpha, Delta, and Omicron). Figure 3 compares the AUC, true positive rate, and true negative rate—metrics not affected by the prevalence of the positive class—of the 5 models per variant.

No clear winner emerged among the models. The global model performs better in predicting mortality for the latest variants but performs worse in predicting ICU admission. This behavior is also observed in the Interpretation section.

**Figure 4:**
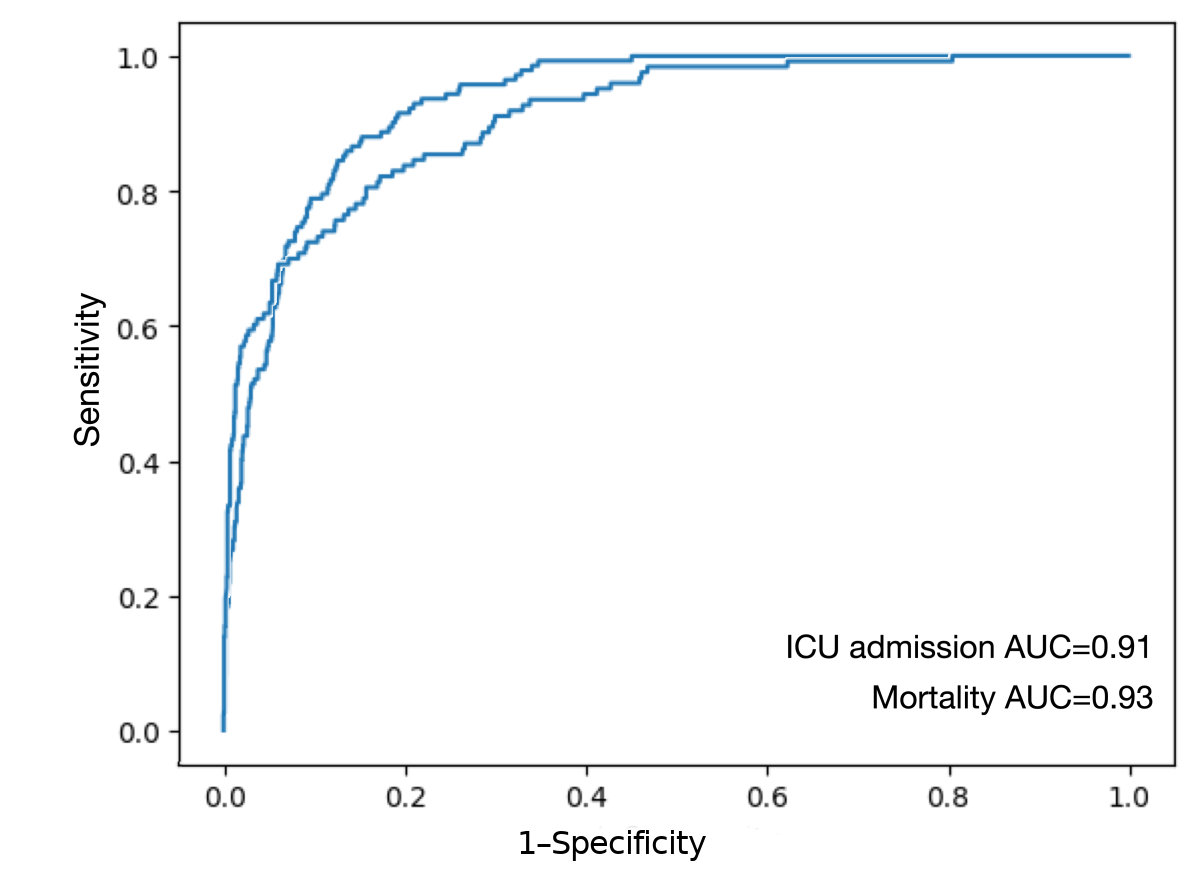
Global predictors ROCs for mortality and ICU admissions.

Since models perform similarly, we present the detailed test metrics for the global model across all variants after evaluating thirty 10-fold cross-validations. The exitus predictor has a mean AUC of 0.92 (SD 0.01), mean best threshold of 0.14 (SD 0.03), mean sensitivity of 0.89 (SD 0.05), mean specificity of 0.82 (SD 0.04), mean positive predictive value (PPV) of 0.35 (SD 0.04; 134/439), and mean negative predictive value (NPV) of 0.98 (SD 0.06; 956/974). ICU predictor has a mean AUC of 0.89 (SD 0.01), mean best threshold of 0.14 (SD 0.05), mean sensitivity of 0.75 (SD 0.05), mean specificity of 0.88 (SD 0.05), mean PPV of 0.37 (SD 0.10; 92/223), and mean NPV of 0.98 (SD 0.01; 1242/1275). Figure 4 presents the receiver operating characteristics (ROCs) of both predictors.

Notably, both predictors exhibit very high NPVs, indicating effective classification for most (>90%) hospitalized patients. In addition, PPVs and specificities allow for the detection of patients who will not die or require ICU admission, advising clinicians to pay more attention to those patients who could do it. These results are consistent regardless of the predominant SARS-CoV-2 variant.

On average, across all model evaluations, the mortality prediction requires at least 70 trees returning a positive outcome to classify a case as positive, whereas the ICU prediction requires 94 trees to do the same. The lower number of features needed for mortality prediction (35 features) compared to ICU prediction (61 features) aligns with this finding (Figure 2).

### Model Validation

Model validation using new records confirms the performance of the global models. With validation hospitalized dataset, the exitus global model has a sensitivity of 0.86, specificity of 0.74, PPV of 0.24 (18/79), NPV of 0.98 (160/163), and AUC of 0.85. The ICU global predictor has a sensitivity of 1.00, specificity of 0.59, PPV of 0.05 (5/106), NPV of 1.00 (0/151), and AUC of 0.82.

With validation nonhospitalized dataset, the exitus global model has sensitivity of 0.88, specificity of 0.98, PPV of 0.21 (30/113), NPV of 0.99 (4/6590), and AUC of 0.95. Especially relevant is that the model was able to predict the mortality of 30 of 34 patients who were not hospitalized.

### Interpretation

#### Mortality

**Figure 5:**
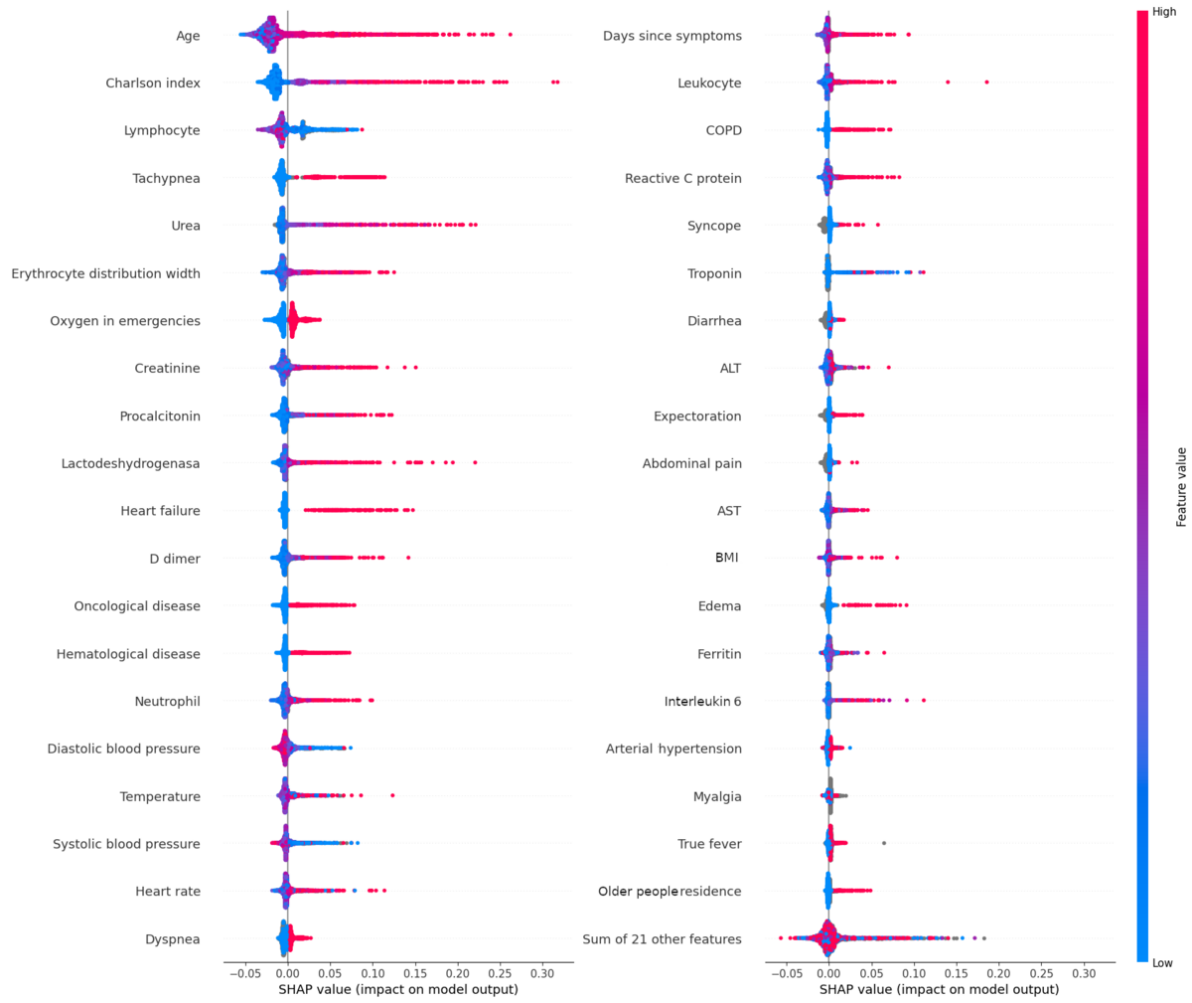
Detailed SHAP values of the 40 most important variables for the exitus model prediction. The red color means a high variable value, whereas the blue one means a low value. Positive SHAP values mean a high impact on the positive of the instance. ALT: alanine aminotransferase; AST: aspartate aminotransferase; COPD: chronic obstructive pulmonary disease; SHAP: Shapley Additive Explanations.

Figure 5 presents the SHAP value of the top 40 features for the prediction of a patient’s mortality. Age, Charlson value, and tachypnea symptoms are the most important features, and the higher their values, the worse the prognosis, whereas low values are protective. High values in urea, erythrocyte distribution width (EDW), oxygen demand in emergencies, creatinine, procalcitonin, lactate dehydrogenase (LDH), heart failure, D-dimer, oncological and hematological diseases, neutrophil, and heart rate indicate a worse prognosis, while higher values of lymphocytes and systolic and diastolic blood pressures are associated with a better prognosis.

An age cohort analysis concludes that a high SHAP value has a greater impact on patients aged 65 years and older. The importance of vaccination is evaluated with a 3-cohort analysis (0, 1, and 2 doses). The results are congruent with the fact that not having been vaccinated or receiving only 1 dose of vaccine is less protective than being completely vaccinated (in our series, only 17 patients have received the Ad26.COV2.S vaccine [Janssen vaccine], and of them, only 10 have received only 1 dose of vaccine).

To evaluate features’ interactions, 60×60 analyses were performed using the SHAP interaction values. We reduce this number by focusing on those with a peak SHAP value above 0.15 (the first 24 features in Figure 5 that account for up to 80% of the mean SHAP value) and when the interaction peak value among the features accounts for more than the 15% of the overall autointeraction SHAP value of the feature. Table 2 presents the interactions that meet the selection criteria.

**Table 2:** Most important interactions between features for mortality prediction.

Feature	Interaction with
Age	Dyspnea
Age	Heart rate
Age	Systolic blood pressure
Charlson	Oncological disease
Neutrophil	Leukocyte

**Figure 6:**
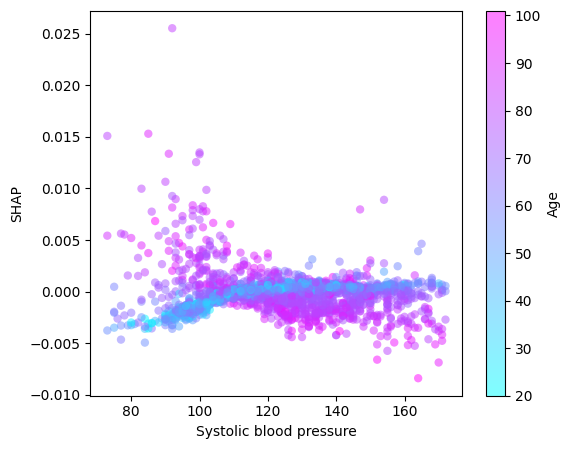
Systolic blood pressure (mm Hg) and age (years) interactions for mortality prediction measured using the SHAP interaction metric. SHAP: Shapley Additive Explanations.

Figure 6 presents the interaction between systolic blood pressure and age: systolic blood pressure above ≈100 mm Hg is protective in older people and below 100 mm Hg is protective for young people.

The SHAP values of the 10 most important features are analyzed over time to detect variations due to changes in the dominant SARS-CoV variant. No significant changes are observed, consistent with the fact that customized models per variant do not lead to performance improvements.

#### ICU Admissions

**Figure 7:**
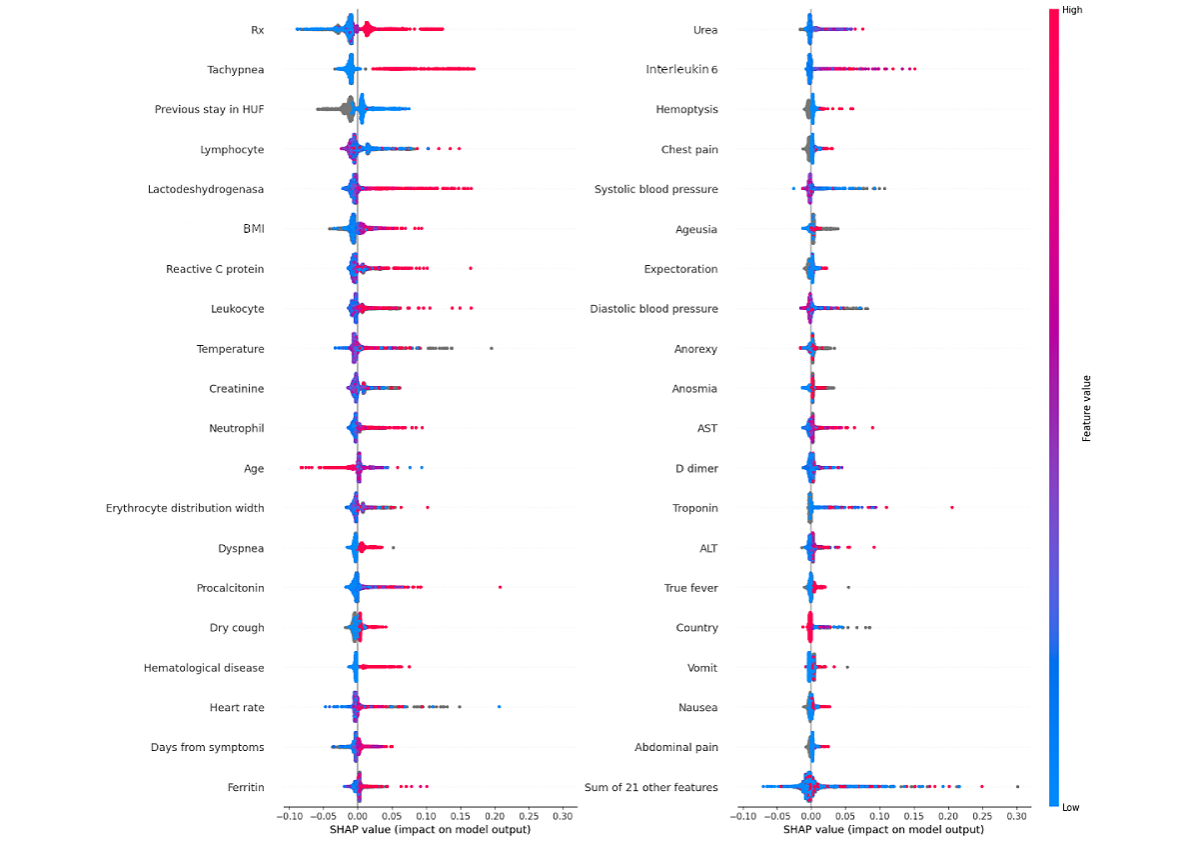
Detailed SHAP values of the most important features for the ICU admission prediction. The red color means a high value of the feature, whereas the blue one means a low value. Positive SHAP values mean a high impact on the positive of the instance. ALT: alanine aminotransferase; AST: aspartate aminotransferase; HUF: Hospital Universitario de Fuenlabrada; Rx: radiography; SHAP: Shapley Additive Explanations.

Figure 7 displays the SHAP values of the top 40 features for predicting ICU admission. The most important features are a high extension in pulmonary infiltrate in the radiology report, tachypnea, LDH, BMI, C-reactive protein, and leukocyte.

**Figure 8:**
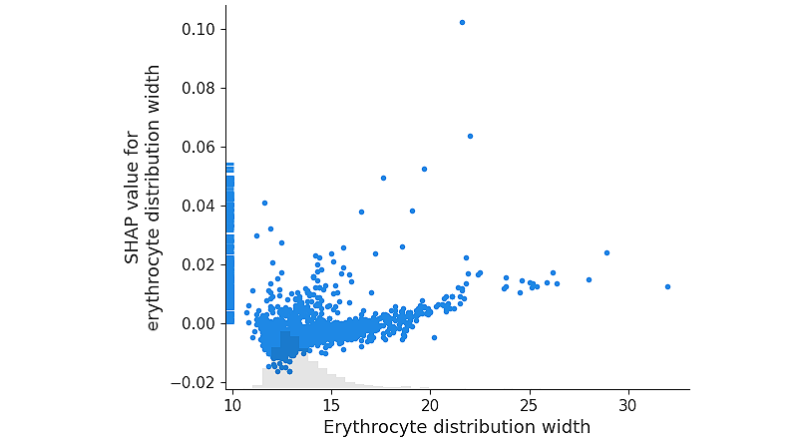
SHAP values for erythrocyte distribution width (in percentages) in the ICU dataset. SHAP: Shapley Additive Explanations.

Features such as creatinine, EDW, and procalcitonin have ambiguous interpretations, as both low and high values contribute to the prediction. Figure 8 provides an example of this behavior for EDW.

Age and vaccination status are not included in the top 10 features, congruent with previous cohorts’ analysis and with the fact that a high ratio of the oldest patients were not admitted to the ICU in the first wave when the median patient’s average age in the ICU was 65 (SD 14) years. This means that not all the patients who died were admitted to the ICU. This was more evident during the first wave when older people in their 80s were not admitted to the ICU due to a lack of available beds, as the health care system was overwhelmed.

We define the critical interactions between features as those with a mean absolute SHAP value greater than 0.03 (the top 30 features in Figure 7), which together account for 80% of the total mean SHAP value. Additionally, the mean absolute interaction value between these features must contribute more than 15% of the feature’s own autointeraction SHAP value.

**Figure 9:**
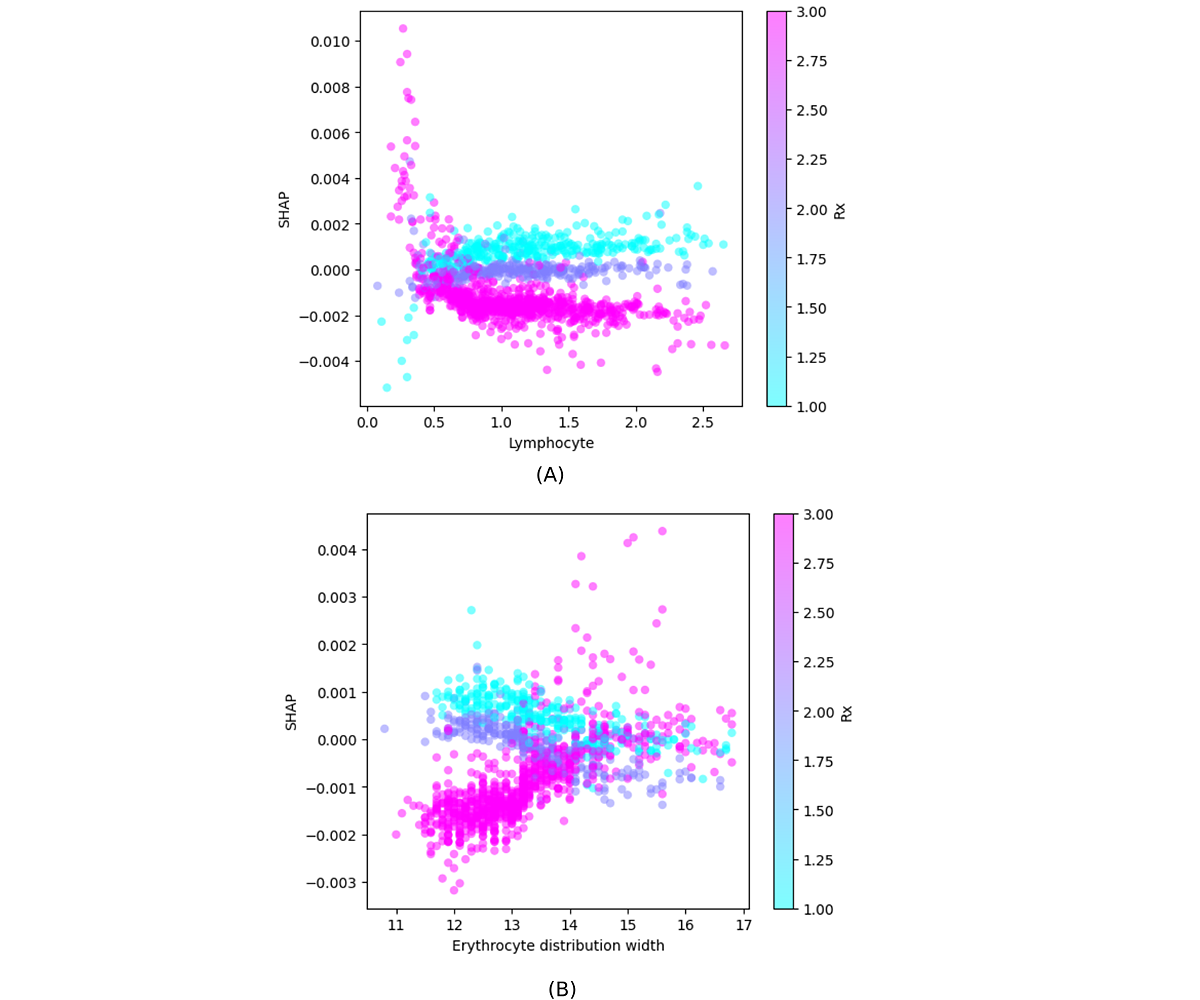
Feature interactions measured using the SHAP interaction metric. (A) Interaction between lymphocyte count (mL–1) and pulmonary infiltrate as observed on Rx (1 means no infiltrate, 2 is unilateral infiltrate, and 3 is bilateral infiltrate). (B) Interaction between erythrocyte distribution width (in percentages) and pulmonary infiltrate (same scale as mentioned earlier). Rx: radiography; SHAP: Shapley Additive Explanations.

Figure 9 exemplifies 2 interactions. Figure 9A illustrates that bilateral pulmonary infiltrate (high values) combined with low lymphocyte counts are associated with an increased probability of ICU admission, but a higher lymphocyte count decreases the ICU admission probability. Figure 9B illustrates a similar behavior for EDW and in combination with pulmonary infiltrate observed on radiography.

## Discussion

### Principal Findings

In this work, we have developed and validated predictive models of mortality and admission to the ICU to be used by clinicians in the first patient assessment using records of nearly 11,000 patients of a population of 220,000 inhabitants in the Fuenlabrada area. We have trained the AI models with nearly 5000 patients who needed hospital admissions throughout the pandemic. Subsequently, we have validated the models using data from over 6000 patients from the city population collected in a different period. Our AI-based models enable us to predict ICU admission and mortality on a case-by-case basis, taking into account different factors such as age, medical history, analytical data, vaccination status, and the date of infection (that indirectly estimates the circulating strain of SARS-CoV-2) at the moment of diagnosis of SARS-CoV-2 infection. The model’s originality is that it can help clinicians to predict “yes or no,” whether a concrete patient will die or need ICU admission at its first evaluation at the hospital.

RF provides a probability score based on the ratio of decision trees that return a positive classification. A threshold value should be provided so that a binary yes or no outcome is returned. The ROC curves in Figure 4 illustrate how the sensitivity and specificity of the model change with the chosen threshold. Therefore, the model does not inherently produce a strict yes or no classification but rather assigns a probability that can be converted into a binary decision based on a predefined threshold. The models in this work use a threshold of 0.14 (Figure 4), determined by the elbow of the ROC curve. This point indicates the optimal trade-off between sensitivity and specificity, making it a reference for selecting the best classification threshold.

There are many pre-existing prediction models for mortality or ICU admission among patients with COVID-19, but most give probabilities and highlight the population characteristics that predict the patient’s outcome. Few of them apply to a specific concrete patient, as our model does. Likewise, our model is also a pandemic and a population model.

Some of the analytical variables we found to be associated with mortality have been described in other studies. Hence, several studies have identified age as the strongest predictor of mortality [23].

An analytical value that deserves to be mentioned is EDW, which is associated with higher mortality in our work. Our findings are consistent with those in the review of Lee et al [24], which included a meta-analysis of 14,866 patients from 10 studies and found that higher levels of EDW were associated with adverse outcomes in patients with COVID-19.

In this sense, there are works, in which red cell distribution width is associated with mortality in patients with COVID-19 who are nonanemic. These associations were independent of age, sex, race, and the Charlson index [25].

Furthermore, we observed a shift toward older patients requiring hospital admission since January 2022, likely due to more than 5,390,000 (80%) of the Madrid population being fully vaccinated, and admissions occurred mainly in older patients with more comorbidities and patients with immunosuppression. While overall admissions decreased, a higher proportion of hospitalizations occurred among individuals aged 65 years and older [26].

Several predictive models related to the severity and prognosis in SARS-CoV-2 infections have been published. Hudda et al [27] reviewed 19 studies, including 7 (37%) focused on model development, 2 (11%) involved external validation of existing models, and 10 (53%) addressed both the development and external validation of the same model.

Asteris et al [28] developed an artificial neural network model based on laboratory indices at admission, achieving high accuracy in predicting ICU hospitalization. They used data from 248 adult patients with COVID-19 for database creation, training, and model development. The 5 most important laboratory indices were the neutrophil to lymphocyte ratio, LDH, fibrinogen, albumin, and D-dimer. The best artificial neural network model achieved an accuracy of 95.97%, a precision of 90.63%, a sensitivity of 93.55%, and an *F*_1_-score of 92.06%, as verified in the validation cohort [28].

Moslehi et al [29] implemented a generalized neural additive model to predict mortality in more than 2000 hospitalized patients. A total of 22 baseline features, including demographic information and clinical biomarkers, were collected. The model achieved an accuracy of 0.84 [29].

Yu et al [30] developed various models to predict in-hospital mortality in patients with COVID-19 during their first day of hospitalization using 138 patient records. Advanced age and the abnormal expression of certain cytokines were found to be closely associated with a worse prognosis. The accuracy of the logistic regression, RF, and decision tree models was 87%, 80%, and 86%, respectively [30].

Our mortality prediction model achieved an AUC of 0.90, with a sensitivity of 0.89, specificity of 0.76, PPV of 0.31, and NPV of 0.98. The model for predicting ICU admission yielded an AUC of 0.89, with a sensitivity of 0.74, specificity of 0.90, PPV of 0.41, and NPV of 0.97. Notably, both models exhibited high NPV, suggesting their effectiveness in identifying patients at low risk of mortality or ICU admission. This is particularly relevant, given that 4238 (90%) of hospitalized patients survived, and 4589 (92%) did not require ICU admission.

Furthermore, the model’s performance is independent of the circulating SARS-CoV-2 variant, demonstrating stability in both testing and external validation. A key advantage is its ability to handle missing values natively, allowing patient classification even when some data are unavailable.

To implement the model into a hospital triage system, an application should be developed that is accessible from the hospital’s digital interface and automatically collects the clinical, analytical, and epidemiological data defined by our model in the evaluation of the patient in the emergency department. This application would perform the calculation used by the model for its prediction, providing an automated response with PPV and NPV, for example, for patient mortality—risk of death: yes or no.

Patients with a no risk of death result could be discharged thinking about the high NPV of the model prediction. Patients with a “yes” risk of death result should be evaluated for hospital admission, or they could be discharged only if they are clinically stable with no supplemental oxygen requirements and with daily clinical follow-up.

This study stands out for its robust validation, which is uncommon among other AI-based predictive models for COVID-19. The validation cohort includes hospitalized patients, potentially infected with Omicron variants, as well as a significant number of patients who were discharged from the emergency department without hospitalization. The model successfully identified most patients who died within 30 days after hospital discharge in the validation cohort (30/34 deaths). We do not know why these patients were not admitted, as hospitalization decisions were made by their personal physicians. Most of these cases occurred during the first wave when the overwhelming health care crisis and epidemic conditions may have influenced such decisions. Nevertheless, we believe this does not impact our analysis, as the model is trained on patients’ initial clinical assessment. As hospitalization decisions are independent of the model, they do not influence its training process or predictions.

### Some Limitations of Our Study

This study is a single-center retrospective study. The results obtained may be influenced by the number of patients hospitalized during each wave and could be specific to periods dominated by a particular SARS-CoV-2 variant. However, our global model has demonstrated stability in these contexts and has also been validated using data from a different period and with patients distinct from those used to create the predictive model.

Another limitation is that our model was developed using data from 5000 patients and validated with another 6000 patients, all belonging to the same region of Spain (the city of Fuenlabrada). It would be of interest to apply this model to other regions in Spain or other countries worldwide to assess its generalizability.

### Conclusions

The scientific community has increasingly focused on integrating AI into COVID-19 predictive modeling. RF techniques were predominantly used in papers published from 2019 to 2023. However, most studies lacked external validation, which hindered reproducibility and applicability to diverse datasets. None of these studies covered the entire pandemic period, and nearly all involved fewer than 5000 patients. While the models demonstrated moderately good performance, with AUC values above 0.7, data collection often included demographics, clinical records, laboratory results, and pharmacological studies, but the applicability settings of these models were often unspecified.

Our study is unique, as it represents a population-based study in a city reflective of the Spanish population. Unlike other studies, it covers a 3-year pandemic period. We developed an AI predictive model for COVID-19–related mortality and ICU admission, which was validated with patients different from those used to create the model. This model covers a 3-year pandemic period, demonstrating its usefulness during both pre- and postvaccination periods. Ethnicity could likely enhance the model’s utility, especially in Southern Europe. We discuss ethnicity because this study has been conducted in a Mediterranean country, specifically in a city with 217,184 inhabitants, where 195,465 (90%) of the population is Spanish and 23,337 (10.7%) are immigrants (n=5991, 2.8% from Central and South America). The model, based on ML, can be automatically applied when a patient seeks medical attention. It is valuable for identifying patients requiring hospitalization and those eligible for home care. This multidisciplinary study, conducted by clinicians and engineers, used data from over 12,000 patients and extensive covariables. Our model offers practical applicability in daily clinical practice, providing a clear risk assessment for mortality and ICU admission.

## Data Availability

Data have not been specifically collected for this study. They are included in a database created for the “Database of COVID-19 patients treated in the Hospital Universitario de Fuenlabrada (FUENCOVID)” project, approved by the Hospital Universitario de Fuenlabrada’s Clinical Research Ethics Committee. Upon manuscript publication, the original dataset used in this paper will be made available after anonymizing the participant data. Data will be freely available to any researcher wishing to use them for noncommercial purposes, without breaching participant confidentiality. The dataset will be accessible after emailing any of the 2 principal coauthors (ogarnica@ucm.es or jose-manuel.ruiz@salud.madrid.org).

## Abbreviations

AI: artificial intelligence
AUC: area under the receiver operating characteristic curve
EDW: erythrocyte distribution width
HUF: Hospital Universitario de Fuenlabrada
ICU: intensive care unit
LDH: lactate dehydrogenase
ML: machine learning
NPV: negative predictive value
PPV: positive predictive value
RF: random forest
ROC: receiver operating characteristic
SHAP: Shapley Additive Explanations
